# Protocol for a systematic review of randomized trials of knee arthroplasty decision aids and shared decision-making approaches

**DOI:** 10.1186/s13643-019-1053-1

**Published:** 2019-06-08

**Authors:** Daniel L. Riddle, Trisha Sando, Talicia Tarver, James Slover, Robert A. Perera, Rafael Sierra, Juan P. Brito, Victor M. Montori

**Affiliations:** 1 Departments of Physical Therapy, Orthopaedic Surgery and Rheumatology, West Hospital, Virginia Commonwealth University, Room B100, 1200 East Broad Street, Richmond, VA 23298-0224 USA; 2 Virginia Commonwealth University, Richmond, VA USA; 3 New York University, Langone Medical Center, New York, NY USA; 4 Mayo Clinic, Rochester, MN USA

**Keywords:** Knee, Arthroplasty, Shared decision-making

## Abstract

**Background:**

Shared decision-making is an approach to making treatment-based decisions that rely on the patient encounter and clear discussions between the patient and the healthcare provider. Patients with arthritis of the knee frequently seek care, and depending on arthritis severity and impact on daily life, joint arthroplasty may be considered as a treatment option. We will conduct a systematic review of shared decision-making trials in knee arthroplasty to determine the types of shared decision-making approaches used and their impact on care received.

**Methods:**

Our systematic review will describe and critically appraise shared decision approaches used in randomized trials of patients undergoing knee arthroplasty, the types of outcomes reported, and the impact of these approaches on the patients’ care. We will use the following databases: PubMed, Web of Science, Embase, CINAHL, PsycINFO, and the Cochrane Library, from inception through December 2018. Additionally, we will assess ongoing research by querying experts and searching trial registries.

**Discussion:**

This study will characterize shared decision-making (SDM) approaches in knee arthroplasty randomized clinical trials and will summarize their effects of SDM on clinical and patient-reported outcomes. We anticipate this review will bring to light knowledge gaps and inform further research into the design and use of shared decision-making approaches in lower extremity arthroplasty.

**Systematic review registration:**

PROSPERO CRD42019123586

**Electronic supplementary material:**

The online version of this article (10.1186/s13643-019-1053-1) contains supplementary material, which is available to authorized users.

## Background

Total knee arthroplasty (TKA) is the most common major surgical procedure conducted in the USA. Approximately one million persons underwent TKA in the USA in 2015, double the volume as compared to 2005 [1]. Demand is likely to continue to increase given that the population of middle-aged and older persons is increasing [2] and, on average, gaining weight [3].

Patient selection for TKA is driven primarily by the surgeon’s assessments of a patient’s knee arthritis severity, pain intensity, and related limitations in addition to surgical fitness as well as the patient’s willingness to undergo the procedure. The Centers for Medicare and Medicaid Services (CMS) has recently mandated a shared decision-making (SDM) approach for some cardiovascular and cancer procedures [4], and these mandates could eventually be applied to other procedures including TKA. Professional organizations like the American Academy of Orthopaedic Surgeons (AAOS) endorse a SDM approach for patients being considered for TKA. The AAOS position statement entitled Shared Physician-Patient Responsibilities [5] reads, “The orthopaedic surgeon should engage in informed shared decision making with the patient using the patient’s values and respect the patient’s decision even if it is in disagreement with the physician’s recommendation.” The AAOS does not define procedures for applying SDM to clinical practice.

An SDM interaction between a patient and a healthcare provider has been defined as having six key elements [6]. The first element is labeled “situation diagnosis” and requires the clinician to understand the patient’s health-related situation and identify aspects of the situation that require action. The second element, “choice awareness,” involves making the patient aware of the various options to address the health-related situation while clarifying the importance of patient preference. The conversation then emphasizes the various options (i.e., “option clarification”) and how these options and their associated benefits and harms align with patient preferences (i.e., “discussion of harms and benefits”). The patient and provider are then in a position to discuss how the various options fit with the patient’s preferences (i.e., “deliberation of patient preferences”). In the final step (i.e., “making the decision”), the patient and clinician together reach a decision for managing the health-related situation. The three-talk model for SDM [7] includes a very similar sequence of elements as described above. Systematic reviews of randomized trial evidence suggest that the impact of SDM on patient cognitive outcomes such as knowledge, affect, and attitude is generally enhanced and decisional conflict is reduced relative to usual care. Health and behavioral outcomes such as self-rated health and biological measures are generally less influenced by SDM and vary depending on the study [8, 9].

Decision aids are tools that assist in some aspects of the shared decision-making process. A decision aid (DA) usually contains content of relevance to a particular clinical decision but typically is delivered to the patient prior to the encounter with the clinician and does not include additional elements of SDM that occur during the encounter. In particular, DAs do not typically extend beyond content related to the disorder or treatments of interest and potential benefits and harms related to a healthcare choice for the disorder of interest. For example, in the TKA literature, Bozic and colleagues used a DA developed by the Informed Medical Decision Foundation to determine if the use of the DA improved patients’ knowledge regarding hip or knee osteoarthritis and their stage of decision-making [10]. Patients randomly assigned to the DA arm were given a digital video disc (DVD) and booklet prior to their surgeon visit. The DVD and booklet described knee OA natural history as well as content describing risks and benefits of surgical and non-surgical options for osteoarthritis. Additionally, patients had a phone-based conversation with a health coach to prepare a list of questions for the surgeon. The DA did not include content related to whether or how the surgeon considered patient preferences when making a TKA recommendation or guidance for either the patient or surgeon regarding the conversation during the encounter. DAs also have been shown to impact cognitive outcomes such as knowledge or decisional conflict relative to usual care [11].

We found no systematic reviews of the TKA DA/SDM literature that specifically determined the types of DA/SDM approaches studied, their relationship to key elements of SDM, or the extent to which DA/SDMs actually impacted the care that the patients received. The purposes of this protocol are to (1) identify and critically appraise the TKA randomized trial literature that has examined the impact of DA/SDM approaches on TKA decisions and outcomes, (2) assess the extent to which the TKA trials of DA/SDM approaches incorporated SDM elements, (3) determine the types of outcomes that were studied and the effects of DA/SDM approaches on these outcomes relative to alternative interventions, and (4) determine the extent to which the DA/SDM approaches used in the trials impacted the care that the patients received. This protocol was modeled after a recently published protocol by Wieringa and colleagues [6].

## Methods

### Study design

Our protocol design was based on the Preferred Reporting Items for Systematic Review and Meta-analysis Protocols (PRISMA-P) [12].

### Study type

Randomized clinical trials will be selected if they compare the use of DA/SDM approaches to either usual care or an active control intervention. No time limit will be established for the search, and all potential study or clinical settings will be included.

### Eligibility criteria

All studies enrolling patients with arthritis and who were scheduled to undergo TKA will be included. No restrictions will be placed on the type of arthritis being treated (e.g., osteoarthritis, rheumatoid arthritis, post traumatic arthritis). Studies that include a mixed sample of patients undergoing either TKA or total hip replacement (THA) will be included only if subgroup data and analyses are provided for patients undergoing TKA. Approximately 1 million patients undergo TKA each year in the USA [1], and a substantial number of TKAs are conducted worldwide. Additionally, the outcomes, recovery trajectories, and patient perceptions of recovery are different for TKA versus THA, and therefore, the focus of this systematic review is on TKA.

### Interventions and comparators of interest

All studies that randomize patients who plan to undergo TKA to a DA/SDM treatment arm and a usual care or active control arm will be included. No restrictions will be placed on the type of usual care or active control that is provided. There also will be no restriction on the type of TKA surgical procedure or the experience of the surgeon conducting the surgery. Patients undergoing either TKA or unicompartmental knee arthroplasty will be included. Data will be disaggregated for TKA and unicompartmental arthroplasty if reported by the authors.

All interventions will be described including those that aim to support elements of SDM during the encounter as well as DAs used before or during the encounter. Descriptions will summarize the key elements of the SDM approach or DA and any associated interventions such as the use of a health coach to aid in DA content understanding [13]. Alternative interventions may also include general educational content about arthritis or usual care treatment and these will be described. Content included in the DA or SDM approach will be specified. For example, Hawker and colleagues used a DA consisting of a 50-min video and booklets that summarized surgical and non-surgical treatment options and their associated benefits, risks, and probabilities [14].

### Information sources and search strategy

The plan was based on a comprehensive approach to RCT identification using the following databases (and corresponding database platforms): MEDLINE (PubMed), Web of Science (Web of Science Core Collection), Embase (Ovid), CINAHL (EBSCO), PsycINFO (APA PsycNET), and the Cochrane Library (CENTRAL). The search strategy was developed with the assistance of a research librarian (TT). All databases will be searched from inception through December 2018. No restrictions will be based on language. Foreign language RCTs will be translated using Google Translate. Reference lists of included studies will be checked for additional sources. All authors of the papers selected for review will be contacted via email to assess whether additional research is ongoing. Finally, several electronic databases will be reviewed to assess for the presence of ongoing RCTs of DA/SDM approaches in TKA. These databases will include the following: ISRCTN.org, Clinicaltrials.gov, and PROSPERO registry. If multiple articles are published from a single RCT, all will be included to assure coverage of all interim analysis time points. Searches of all databases will be documented in a table to assure complete reporting of all search results. The search strategy and preliminary number of papers meeting the search strategy for each database are presented in Additional file 1.

### Study records

A searching data management software (i.e., Covidence) will be used by two independent reviewers (DLR and TS) to identify and track searches from all relevant databases. The two reviewers will screen all titles and abstracts independently identified from the search strategy for the included databases. Full reports will be obtained for each title/abstract judged to be potentially relevant. Reasons for exclusion will be reported for each database using the PRISMA flow diagram. A third independent reviewer (JS) will resolve any discrepancies resulting from the initial review. The extent of agreement for excluded papers and papers selected for inclusion by the two reviewers will be assessed using the Kappa statistic (*Κ*), an agreement index that accounts for chance agreement [15].

A standardized data extraction table will be used to collect relevant data on each included trial (see Additional file 2). The table was modified based on the recently published data extraction table by Wieringa and colleagues [6]. The data table includes key headings of the publication details, study design, population of interest, sociodemographic characteristics of the samples, inclusion and exclusion criteria, study setting, details of experimental intervention and comparison intervention, duration of follow-up and outcomes studied, and extent of effectiveness for both cognitive and clinical outcomes of the DA/SDM approach in relation to the comparator. In addition, a summary of the six key elements of SDM tools [6] will be collected for each RCT. Particular attention will be paid to the current availability of DA/SDM approaches used in the included studies. If the DA/SDM is commercially available, this will be reported. We will contact the corresponding author of all eligible studies with three purposes: (a) to verify that we have characterized their studies correctly, (b) to complete any missing data, and (c) to ask for any unpublished studies. The method of contact will be via email, sending two email notices separated by 2 weeks. If there is no answer, we will pursue contact of the first author or senior author (if different from the corresponding one) in similar fashion.

### Outcomes and prioritization

All outcomes collected at every time point will be included in the review. The primary outcome of interest will be cognitive/affective outcomes including but not limited to decisional conflict [16], content knowledge regarding arthritis, and treatment decision preference. Secondary outcomes will be clinical outcomes including but not limited to satisfaction with surgical outcome, patient-reported pain and functional outcome, surgical versus non-surgical treatment decisions made during the encounter, and any other clinical outcome data collected in any of the included studies. Either cognitive/affective outcomes or clinical outcomes may be proximal (e.g., measured immediately after the clinician encounter) or distal (e.g., typically measured weeks or months after the encounter) [17].

Outcome measures used in DA studies tend to focus primarily on proximal cognitive/affective outcomes related to whether the patient’s review of the DA content prior to the clinician encounter led to improvements in, for example, decisional conflict or content knowledge. Minimal emphasis is typically placed on the measurement of outcomes of the encounter itself. Common outcome tools in DA studies include but are not limited to the decisional conflict scale [18] and the Hip/Knee Osteoarthritis Decision Quality Instrument a scale, an example of a content knowledge survey [19]. In contrast, SDM studies place major focus on the clinical encounter and whether the SDM tool led to clinical decisions and interactions that facilitated and were consistent with patient preferences. Common outcome tools in SDM include but are not limited to the SDM-Q-9 [20] and the OPTION scale [21], both of which are used to collect detailed patient/clinician encounter data. Both DA and SDM approaches have included proximal clinical outcomes including satisfaction with the clinical decision as well as distal clinical outcomes such as 30-day post-treatment adverse event rates [22]. Our review will comprehensively assess the outcomes used in the trials and report the extent to which outcomes assess both cognitive/affective outcomes and clinical outcomes at proximal and distal time points as well as the extent to which the outcomes assess the encounter itself and the decision-making process used during the encounter.

### Handling missing data

If data reported in a study is unclear or missing, we will request information from the authors using the approach described above. All attempted contacts will be documented.

### Risk of bias in individual studies

Bias risk for each trial will be assessed by two independent reviewers (DLR and TS) using the risk of bias tool (RoB 2) developed by the Cochrane Collaboration [17]. This bias instrument assesses five domains of potential bias: the randomization process, deviations from intended interventions, outcome data missingness, outcome measurement, and reported result biases. Each domain is assessed using multiple items, and an overall risk of bias is rated as low, high, or some concerns. The Kappa statistic will be used to judge the extent of agreement for each of the six domains, after accounting for chance agreement, by the two raters. If agreement is less than moderate for a domain-specific risk of bias score (i.e., *Κ* = 0.4 or less) [15], a third reviewer (JS) will arbitrate differences until an acceptable level of agreement is achieved.

### Synthesis of study data

We will describe the DA/SDM approaches for each trial in our review as well as the extent to which each trial addressed the 6 key elements of SDM [6] along with cognitive and clinical outcomes of each trial. Additionally, for each trial, we will report the extent to which the DA/SDM arm impacted the actual care that the patients received, as compared to the usual care/active control arm. For continuous outcomes (e.g., decisional conflict scale [16]), mean differences or mean changes between AD/SDM and usual care/active control group, together with *p* values and 95% confidence intervals (95% CIs), will be extracted. For dichotomous outcomes, risk ratios (RRs) or odds ratios (ORs) with 95% CIs will be extracted or calculated if possible. To display heterogeneity and effect sizes, forest plots will be used. A random effects meta-analysis model to account for both between- and within-study variance will be used. The pooled effect size across studies will be estimated using a random effects meta-analysis model. Studies will be weighted by the inverse of the variance of the parameter estimates. The *Q* statistic and *I*^2^ index will be used to assess heterogeneity across studies. Because we expect substantial inconsistency in participants, interventions, comparators, and outcomes, where sensible, we will conduct a random effects meta-regression with the intent of exploring for subgroup interactions between patient DA and conversational SDM approaches. The “metafor” package in the R statistical software [18] will be used to complete all analyses with an alpha of 0.05 [19]. We estimate that all data analyses will be completed by the end of October of 2019. Narrative synthesis may be utilized for those content areas in which only one or very few RCTs have been conducted, for example, trials conducted on persons with unicompartmental knee arthroplasty [21] or in TKA access studies of participants who are African American [22].

Protocol amendments prior to the start of data collection will be made by posting changes to the PROSPERO registry information (see CRD42019123586). Changes made after the start of data collection will be reported in subsequent systematic review publications.

### Meta-biases

To examine potential publication bias, a contour-enhanced funnel plot will be reported [20].

### Confidence in cumulative evidence

The Grading of Recommendations Assessment, Development and Evaluation (GRADE) approach to judging overall quality of evidence and recommendations as defined in the *GRADE Handbook* will be applied to the evidence generated in this systematic review [23]. These recommendations include an overall quality of evidence rating for each outcome as high, moderate, low, or very low. The GRADEpro GDT software will be used to generate GRADE data [24].

## Discussion

This review will provide a comprehensive summary of the clinical trial evidence related to the use of DA/SDM approaches as applied to patients undergoing TKA, the most common major surgical procedure conducted in the USA. To date, several DA/SDM clinical trials are known to have been published [14, 22, 25–28], and it is likely that the comprehensive search planned as part of the systematic review will identify other trials. The findings from this systematic review will inform future research of DA/SDM approaches for patients with TKA at a time when substantial emphasis is being placed on SDM approaches for beneficiaries by third-party payers such as the Centers for Medicare and Medicaid Services [4]. Specifically, this review will inform the extent to which the current DA/SDM conforms to the six key elements of SDM and the extent to which the studies used DA/SDM methods to guide and influence surgeon/patient conversations regarding TKA candidacy. These findings have potential to not only impact future research on SDM methods related to TKA decision-making but may also inform orthopedic surgeons of the practical issues to consider when attempting to incorporate SDM applications in daily practice. Uptake of DA/SDM approaches in daily practice has been limited and has been attributed to a variety of factors including the perception by orthopedic surgeons that patient outcomes were already optimal, a lack of alternatives to TKA, and concerns regarding medico-legal implications of using a DA/SDM tool [29]. Additionally, while some surgeons may be concerned about the additional time and methods required to incorporate SDM approaches to daily care, evidence suggests that the time necessary to incorporate effective contemporary SDM approaches adds only a few minutes to an encounter [30].

There are limitations to our review. Our focus is on RCTs of DA/SDM approaches for TKA given that RCTs are considered the most valid method for determining causal associations between treatment and outcome [31]. However, we may exclude potentially well-described and well-studied DA/SDM approaches applied only in observational studies. Additionally, our focus is on TKA and the results will not directly apply to DA/SDM approaches applied to other orthopedic surgical approaches though the concepts discussed in the review may assist others in applying DA/SDM principles to other surgical approaches in orthopedics including hip arthroplasty and fracture treatment.

## Additional file

Additional file 1: The search strategy and preliminary number of papers 414 meeting the search strategy for each database. (DOCX 18 kb)

Additional file 2: A standardized data extraction table used to collect 415 relevant data on each included trial. (DOCX 26 kb)

## Data Availability

Not applicable.

## Abbreviations

AAOS: American Academy of Orthopaedic Surgeons
CMS: Centers for Medicare and Medicaid Services
DA: Decision aid
DVD: Digital video disc
PRISMA-P: Preferred Reporting Items for Systematic Review and Meta-analysis Protocols
SDM: Shared decision-making
THA: Total hip arthroplasty
TKA: Total knee arthroplasty
*Κ*: Kappa statistic
