# Supplementary material for: Protocol for a systematic review of randomized trials of knee arthroplasty decision aids and shared decision-making approaches
Source: Syst Rev. 2019 Jun 8;8:137. doi: 10.1186/s13643-019-1053-1 (PMC6555949; doi:10.1186/s13643-019-1053-1)
Supplement: Supplementary file 1 — The search strategy and preliminary number of papers 414 meeting the search strategy for each database. (DOCX 18 kb) [file 13643_2019_1053_MOESM1_ESM.docx]

Additional file 1

**Search Strategy**

**Systematic Review of Randomized Trials of Knee Arthroplasty Decision Aids and Shared Decision-Making Approaches**

***PubMed: from inception until December 31, 2018***

((("Decision Making"[Mesh] OR "Clinical Decision-Making"[Mesh] OR Decision*[tiab] OR "Patient-Centered Care"[Mesh] OR "Patient Participation"[Mesh] OR "Patient Preference"[Mesh] OR share[tiab] OR shared[tiab] OR patient centered*[tiab] OR patient centred[tiab] OR patient focused[tiab] OR sdm[tiab] OR preference[tiab] OR preferences[tiab]) OR "Decision Support Techniques"[Mesh] OR "Decision Making, Computer-Assisted"[Mesh] OR tool*[tiab] OR aid[tiab] OR aids[tiab] OR intervention*[tiab] OR support*[tiab] OR instrument*[tiab] OR "Checklist"[Mesh]))

AND (“Arthroplasty, Replacement, Knee"[Mesh] OR "Knee Joint/surgery"[Mesh] OR knee replacement[tiab] OR knee replacements[tiab] OR tkr[tiab] OR tka[tiab]))

AND (((clinical[Title/Abstract] AND trial[Title/Abstract]) OR clinical trials as topic[MeSH Terms] OR clinical trial[Publication Type] OR random*[Title/Abstract] OR random allocation[MeSH Terms] OR therapeutic use[MeSH Subheading]))

**Results: 2922**

***CINAHL: from inception until December 31, 2018***

[MH ("Decision Making" OR "Decision Making, Clinical" OR "Decision Making, Patient") OR TI decision* OR AB decision* OR decision making TI OR decision making AB OR

MH ("Patient Centered Care" OR "Consumer Participation") OR TI (share OR shared OR patient centered* OR patient centred OR patient focused OR sdm OR preference OR preferences) OR AB (share OR shared OR patient centered* OR patient centred OR patient focused OR sdm OR preference OR preferences) OR

MH ("Decision Support Techniques+" OR "Decision Support Systems, Clinical" OR "Decision Making, Computer Assisted" OR "Checklists") OR TI (tool* OR aid OR aids OR intervention* OR support* OR instrument*) OR AB (tool* OR aid OR aids OR intervention* OR support* OR instrument*) ]

AND

( (MH "Arthroplasty, Replacement, Knee+") OR (MH "Knee Surgery+") ) OR TI ( knee replacement* OR total knee OR tkr OR tka ) OR AB ( knee replacement* OR total knee OR tkr OR tka )

Filters:

Therapy – High Sensitivity

Date range: inception – December 31, 2018

**Results: 3300**

***Cochrane: from inception until December 31, 2018***

(MeSH (Decision Making OR Clinical Decision-Making OR Decision Making, Computer-Assisted) OR decision* OR decision making) OR (MeSH (Patient-Centered Care) OR share OR shared OR patient centered* OR patient centred OR patient focused OR sdm OR preference OR preferences) OR (MeSH (Checklists) OR tool* OR aid OR aids OR intervention* OR support* OR instrument*)

AND

MeSH (Arthroplasty, Replacement, Knee) OR (knee surgery OR total knee replacement* OR knee replacement* OR tkr OR tka

AND

rct OR random* OR control* OR trial OR placebo* OR compar* OR group OR groups OR therapy OR treatment OR intervention OR “research design” OR comparative OR “evaluation stud*” OR “follow-up stud*” OR prospective OR “single blind” OR “double blind” OR “trebl* blind” OR “triple blind” OR factorial OR allocat* OR assign* OR volunteer* OR crossover OR “cross over”

**Results: 2928 total; 2860 Trials**

***Embase: from inception until December 31, 2018***

['decision making'/exp OR decision making :ab,ti OR decision*:ab,ti OR

'patient care'/exp OR 'patient participation'/exp OR 'patient preference'/exp OR share:ab,ti OR shared:ab,ti OR ‘patient centered*’:ab,ti OR ‘patient centred’:ab,ti OR ‘patient focused’:ab,ti OR sdm:ab,ti OR preference:ab,ti OR preferences:ab,ti OR

'checklist'/exp OR 'decision support system'/exp OR tool*:ab,ti OR aid:ab,ti OR aids:ab,ti OR intervention*:ab,ti OR support*:ab,ti OR instrument*:ab,ti OR checklist*:ab,ti]

AND

'total knee arthroplasty'/exp OR total knee replacement/exp OR total knee replacement*: ab,ti OR tkr: ab, ti OR tka:ab,ti

AND

Random.tw. OR clinical trial.mp. OR ‘health care quality’/exp

**Results: 2255**

***Web of Science: from inception until December 31, 2018***

[TS = (Decision* OR clinical decision making OR shared decision making OR decision making) OR

TS = (share OR shared OR patient centered* OR patient centred OR patient focused OR sdm OR preference OR preferences) OR

TS = (tool* OR aid OR aids OR intervention* OR support* OR instrument* OR checklist*)]

AND

TS = (knee arthroplasty OR knee joing surgery OR knee replacement* OR total knee replacement* OR tkr OR tka)

AND

TS = (rct OR random* OR control* OR trial OR placebo* OR compar* OR group OR groups OR therapy OR treatment OR intervention OR “research design” OR comparative OR “evaluation stud*” OR “follow-up stud*” OR prospective ” OR “single blind” OR “double blind” OR “trebl* blind” OR “triple blind” OR factorial OR allocat* OR assign* OR volunteer* OR crossover OR “cross over”) OR TI= (rct OR random* OR control* OR trial OR placebo* OR compar* OR group OR groups OR therapy OR treatment OR intervention OR “research design” OR comparative OR “evaluation stud*” OR “follow-up stud*” OR prospective ” OR “single blind” OR “double blind” OR “trebl* blind” OR “triple blind” OR factorial OR allocat* OR assign* OR volunteer* OR crossover OR “cross over”)

**Results: 8638**

***PsycINFO: from inception until December 31, 2018***

[DE "Decision Making" OR TI decision* OR AB decision* OR TI decision making OR AB decision making) OR

DE ("Client Participation" OR "Caring Behaviors) OR TI (share OR shared OR patient centered* OR patient centred OR patient focused OR sdm OR preference OR preferences) OR AB (share OR shared OR patient centered* OR patient centred OR patient focused OR sdm OR preference OR preferences) OR

DE ("Decision Support Systems" OR "Cheklist (Testing)) OR TI (tool* OR aid OR aids OR intervention* OR support* OR instrument* OR checklist*) OR AB (tool* OR aid OR aids OR intervention* OR support* OR instrument* OR checklist*)]

AND

knee arthroplasty OR knee joint surgery OR knee replacement* OR total knee replacement* OR tkr OR tka

**Results: 264**
