# Supplementary material for: Protocol for a systematic review of randomized trials of knee arthroplasty decision aids and shared decision-making approaches
Source: Syst Rev. 2019 Jun 8;8:137. doi: 10.1186/s13643-019-1053-1 (PMC6555949; doi:10.1186/s13643-019-1053-1)
Supplement: Supplementary file 2 — A standardized data extraction table used to collect 415 relevant data on each included trial. (DOCX 26 kb) [file 13643_2019_1053_MOESM2_ESM.docx]

Additional file 2

Data Extraction - **Systematic Review of Randomized Trials of Knee Arthroplasty Decision Aids and Shared Decision Making Approaches**

**REFERENCE**

1) Full Title:

2) Authors (surname 1^st^ author, first initial 1^st^ author, second initial 1^st^ author, surname

2^nd^ author, etc):

3) Publication:

4) Volume (Issue):

5) Page numbers (if available):

6) Year of Publication:

7) Language of original full text article (select one):
 English Spanish German Other:

8) Is the paper an:

RCT Pilot RCT

9) Funding Source:

**STUDY DESIGN**

*Inclusion Criteria*

10) Describe all inclusion criteria:

*Exclusion Criteria*

11) Describe all exclusion criteria:

*Study Design*

12) The study was conducted as a:

Individual randomization design

Cluster randomization design

Cross-over design

*Trial Setting*

13) The trial was conducted in:

Community-based setting

Academic medical center setting

Combination of the two

Not specifically stated

*Study Population*

14) Total number of participants included:

15) Total number of participants analyzed:

Analyzed per protocol:

Analyzed by intent to treat:

16) Level of education of study population:

17) Socioeconomic level of study population:

18) Surgical intervention:

Total knee arthroplasty (TKA)
 Unicompartmental knee arthroplasty

Total hip arthroplasty (THA)

19) Country where RCT took place:

20) Mean age and standard deviation:

21) Percentage females:

*Comparison(s)/Control Intervention(s)*

22) Comparison(s)/control intervention(s) (check one or more boxes):

Usual care (define usual care if provided):

Active control, namely (provide description):

*Follow-up Period*

23) Number of measurement sessions, including baseline and all follow-up:

24) Time between each measurement session:

*Outcomes Studied*

25) Check all types of outcomes included in the study (check one or more):

Cognitive/affective outcomes (list each outcome measure)

………………………………………………

………………………………………………

………………………………………………

Clinical outcomes (list each outcome measure)

………………………………………………

………………………………………………

………………………………………………

**CHARACTERISTICS OF THE INTERVENTION**

*Description of the Intervention*

26) Name of the intervention:

27) Setting in which the intervention is meant to be used (check one or more boxes):

At the patient’s home

In the clinic waiting room

In the clinic room

Setting not reported

28) Timing of intervention use (check one or more boxes):

Pre-encounter with surgeon

Encounter with surgeon

Post-encounter with surgeon

Timing not reported

29) Intervention used by (check one or more boxes):

Clinician- specify type (check one or more boxes):

Surgeon

Nurse

Clinical Specialist (name type):

Other: ……………………………………………………………….

Research personnel

Patient

Relative of patient

Other: ……………………………………………………………….

30) Mode of delivery for the intervention:

Online/digital

Physical (paper, etc)

Both online/digital and physical available
 Other: ……………………………………………………………….

31) Is clinical training required to use the intervention?

Yes No

32) Description of the intervention, be as specific as possible:

33) Key elements of SDM interaction:

a) Situation diagnosis:

Yes No Unable to determine

a) Choice awareness:

Yes No Unable to determine

a) Option clarification:

Yes No Unable to determine

a) Discussion of harms and benefits:

Yes No Unable to determine

a) Deliberation of patient preferences:

Yes No Unable to determine

a) Making the decision:

Yes No Unable to determine

*Intervention Development and Funding Source*

34) Who developed the intervention:

35) Is the intervention still available?

Yes No

36) If the intervention is available, provide website, location, etc. to be used to obtain the intervention:

Intervention not available

**STUDY MEASUREMENTS**

Complete this section for each type/target of measurement taken in the study

*Measurement Instrument*

37) Target of measurement (check one):

Patient

Clinician

Patient-clinician interaction/conversation

System

38) Type of cognitive/affective outcome measured:

Decisional conflict

→ measurement instrument used: ………………………………………………

Knowledge regarding arthritis

→ measurement instrument used: ………………………………………………

Treatment decision (preference)

→ measurement instrument used: ………………………………………………

Treatment satisfaction

→ measurement instrument used: ………………………………………………

Decision satisfaction

→ measurement instrument used: ………………………………………………

Conversation satisfaction

→ measurement instrument used: ………………………………………………

Risk expectations and perceptions

→ measurement instrument used: ………………………………………………

Other:

→ measurement instrument used: ………………………………………………

39) Type of clinical outcome measured:

Decision to have surgery → yes/no

Total knee arthroplasty utilization

→ measurement instrument used: ………………………………………………

Satisfaction with surgical outcome

→ measurement instrument used: ………………………………………………

Pain level

→ measurement instrument used: ………………………………………………

Functional outcome

→ measurement instrument used: ………………………………………………

Strength

→ measurement instrument used: ………………………………………………

Range of motion

→ measurement instrument used: ………………………………………………

Achieving treatment goal

→ measurement instrument used: ………………………………………………

Other:

→ measurement instrument used: ………………………………………………

*Study Results*

40) For each continuous outcomes; 95%-CI, point estimate/beta, p-value:

………………………………………………

………………………………………………

………………………………………………

41) For each dichotomous or ordinal outcomes: 95% CI, OR (if RR provided then calculate OR), p-value:

………………………………………………

………………………………………………

………………………………………………
